# MR‐guided pulsed focused ultrasound improves mesenchymal stromal cell homing to the myocardium

**DOI:** 10.1111/jcmm.15944

**Published:** 2020-10-17

**Authors:** Kee W. Jang, Tsang‐Wei Tu, Robert B. Rosenblatt, Scott R. Burks, Joseph A. Frank

**Affiliations:** ^1^ Frank Laboratory, Radiology and Imaging Sciences National Institutes of Health Bethesda MD USA; ^2^ Office of Product Evaluation and Quality Center for Devices and Radiological Health U.S. Food and Drug Administration Silver Spring MD USA; ^3^ Molecular Imaging Laboratory Department of Radiology Howard University Washington DC USA; ^4^ Center for Neuroscience and Regenerative Medicine Uniformed Services University of the Health Sciences Bethesda MD USA; ^5^ National Institute of Biomedical Imaging and Bioengineering National Institutes of Health Bethesda MD USA

**Keywords:** enhanced homing permeability and retention, magnetic resonance imaging, mesenchymal stromal cells, myocardium, pulsed focused ultrasound

## Abstract

Image‐guided pulsed focused ultrasound (pFUS) is a non‐invasive technique that can increase tropism of intravenously (IV)‐infused mesenchymal stromal cells (MSC) to sonicated tissues. MSC have shown promise for cardiac regenerative medicine strategies but can be hampered by inefficient homing to the myocardium. This study sonicated the left ventricles (LV) in rats with magnetic resonance imaging (MRI)‐guided pFUS and examined both proteomic responses and subsequent MSC tropism to treated myocardium. T2‐weighted MRI was used for pFUS targeting of the entire LV. pFUS increased numerous pro‐ and anti‐inflammatory cytokines, chemokines, and trophic factors and cell adhesion molecules in the myocardial microenvironment for up to 48 hours post‐sonication. Cardiac troponin I and N‐terminal pro‐B‐type natriuretic peptide were elevated in the serum and myocardium. Immunohistochemistry revealed transient hypoxia and immune cell infiltration in pFUS‐targeted regions. Myocardial tropism of IV‐infused human MSC following pFUS increased twofold‐threefold compared with controls. Proteomic and histological changes in myocardium following pFUS suggested a reversible inflammatory and hypoxic response leading to increased tropism of MSC. MR‐guided pFUS could represent a non‐invasive modality to improve MSC therapies for cardiac regenerative medicine approaches.

## 1 INTRODUCTION

Pulsed focused ultrasound (pFUS) has been increasingly studied as a non‐invasive technique that can serve as an adjunct to enhance cellular therapy in treatment of disease.^1^, ^2^, ^3^ The mechanotransductive effect of pFUS has been shown to induce transient local molecular changes in expression of cytokines, chemokines and trophic factors (CCTF), as well as cell adhesion molecules (CAMs) without damage to the targeted tissue.^4^, ^5^, ^6^, ^7^, ^8^ The microenvironmental changes in CCTF and CAM following pFUS have been demonstrated to increase homing of mesenchymal stromal cells (MSC) to a variety of normal and diseased tissues.^4^, ^5^, ^6^, ^8^, ^9^, ^10^, ^11^, ^12^, ^13^ Furthermore, delivery of MSC represents a promising therapy for a variety of cardiac pathologies, especially infarcted myocardium^14^, ^15^; however, the infusion of these cells often demonstrates inefficient myocardial homing and requires invasive techniques for adequate delivery.

pFUS has been applied for various cardiac applications such as treating cardiac arrhythmias, as a cardiac pacing tool, as a contusion model and as a method to mitigate cardiac diseases.^16^, ^17^, ^18^, ^19^ The current study investigates whether pFUS could be used to modify the myocardial microenvironment and increase tropism of IV‐injected MSC. Ultrasound exposures have previously demonstrated increased stem cell homing to myocardium, but these indications coupled with intravenous microbubble (MB) infusion can cause ultrasound‐targeted MB destruction (UTMB).^17^, ^18^, ^19^ UTMB has been shown to cause some molecular changes that are necessary to induce MSC tropism, but UTMB often results in tissue damage making the approaches less attractive as a regenerative medicine technique.

In this study, pFUS without MB was administered to the left ventricle at parameters previously shown not to damage other tissues. We investigated whether pFUS could generate cellular and molecular changes in the heart to ultimately enhance permeability and retention of human mesenchymal stromal cells (MSC) in treated myocardium. The pFUS treatments presented here could provide clear benefit to non‐invasively increasing MSC homing to myocardium without causing tissue damage associated with UTMD.

## 2 MATERIALS AND METHODS

### 2.1 Animals

All animal experiments were approved by the Animal Care and Use Committee at the NIH Clinical Center and were performed in accordance with the National Research Council's Guide for the Care and Use of Laboratory.^20^ Eight‐to‐ten‐week‐old female Sprague Dawley rats (Charles River Laboratories, Wilmington, MA) were provided free access to food and water during the study. The hair on the chest was removed with depilatory cream prior to pFUS treatment, and the average weight of rats was 230.2 ± 9.7 g.

### 2.2 MR‐guided pFUS and MRI

**Figure 1:**
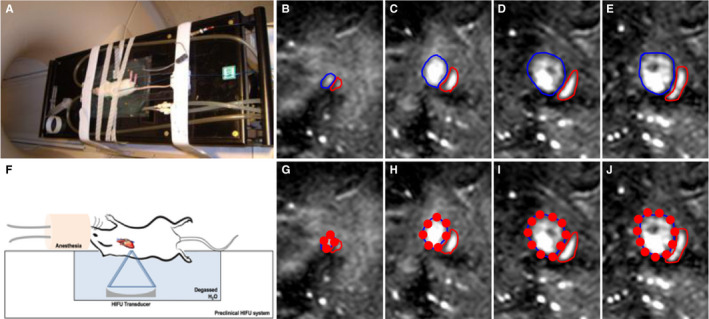
MR‐guided pFUS. A, Rats placed on a MR‐compatible pre‐clinical focused ultrasound system. F, Illustration of experimental setup of pFUS treatment to the rat heart. Left side of the rat chest was submerged in degassed water to pose the heart perpendicular to the focused ultrasound transducer. B‐E, Sequential T2w coronal MR images were acquired at 1 mm slice thickness for pFUS to target the left ventricle of the rat heart. Blue outline area represents the left ventricle, and red outline area represents the right ventricle. G‐J, pFUS targeting spots in red circle based on the MR guidance images

Rats (n = 87) were placed on a pre‐clinical MR‐compatible image‐guided high intensity focused ultrasound (HIFU) system (RK‐100, FUS instruments, Ontario, Canada). The left side of the chest submerged in degassed H_2_O maintained at 37°C in order to place the heart perpendicular to the ultrasonic transducer (Figure 1A). The rats were anesthetized with 1.5% of isoflurane in 100% O_2_ during the pFUS treatment (Figure 1).

MR images performed with multi‐slice fast field echo (FFE) sequences with repetition time (TR) = 8.9 ms, echo time (TE) = 4.5 ms, flip angle (FA) = 45° and a slice thickness of 1 mm with an in‐plane resolution of 0.14 × 0.14 mm^2^ of the heart were acquired using a clinical 3T scanner with a radiofrequency receive coil (Achieva, Philips Healthcare, USA) without cardiac or respiratory gating. A focused ultrasound transducer with a centre frequency = 1.1 MHz, focal length = 60 mm, transducer diameter = 75 mm (F = 0.8) and electro‐acoustic conversion efficiency = 75.9% was used at 3 MPa of peak negative pressure (PNP). The focal dimensions of pFUS beam had a full width half max diameter = 1.15 mm and length = 8.4 mm. Sonication bursts were 10 ms in length with a pulse repetition time of approximately 0.33 Hz depending on number and location of treatment spots determined during treatment planning. Sonications generated 3 MPa of peak negative pressure (PNP) measured in degassed water, which corresponded to ultrasound intensities of 660 W/cm^2^ spatial‐average temporal‐peak intensity [I_SATP_], and approximately 2.2 W/cm^2^ spatial‐average temporal‐average [I_SATA_] based on typical repetition times. Transducer positioning was controlled using an LP100 (FUS Instruments, Toronto, Canada). Under the MRI guidance, approximately 40 sites up to 4mm from the heart apex covering the left ventricle were sonicated with each focal spot receiving 100 pulses (Figure 1C‐J).

### 2.3 Proteomic analysis of myocardium and serum following pFUS

The pFUS‐targeted myocardium and blood were harvested (n = 5‐10 rats/time point) at post‐0 (SHAM (SH) or Baseline (BL)), 0.25, 1, 3, 6, 12, 18, 24, 36, 48 and 96 hours. The harvested myocardium was frozen immediately in liquid nitrogen. The blood was also harvested to analyse the cardiac injury marker expression following pFUS with centrifugation at speed 500g  (3,000 rpm) for 10 minutes to isolate the serum, which was stored in −80°C for further analysis. The SH group was treated with pFUS with 0 watts (PNP = 0 MPa) to the transducer and was termed as 0 hour. The frozen tissues were then homogenized in cell lysis buffer containing a protease inhibitor cocktail (S8820‐2TAB; Sigma‐Aldrich, St. Louis, MO), and the total amount of proteins in the homogenates was determined by bicinchoninic acid assay (23227; Thermo Fisher Scientific, Waltham, MA). The homogenates (2 mg/mL total protein) were analysed by rat Cytokine/Chemokine Magnetic Bead Panel Multiplexed Immunoassay Assay kit (RECYMAG65K27PMX; EMD Millipore, MA), as well as single ELISA kits for cTnI (LS‐F23616, LSBio Inc, Seattle, WA), NT‐proBNP (LS‐F23593, LSBio Inc), TNF‐α (AB46070, Abcam, Cambridge, MA) and IL‐1 alpha (IL1‐α) (AB113350, Abcam) according to the manufacturer's protocols. Multiplex assays were read on a BioPlex 200 (Bio‐Rad, Hercules, CA), and singleplex assays were read on a spectrophotometric plate reader (Spectra Max M5, Molecular Devices, Sunnyvale, CA).

### 2.4 MSC culture and administration

Human MSC (provided and characterized by Bone Marrow Stromal Cells Transplantation Center at our institution) were obtained from volunteers undergoing bone marrow biopsy under an approved institutional review board protocol at our institution (www.clinicaltrials.gov, NCT01071577). MSCs were cultured in a minimum essential medium (a‐MEM) supplemented with 2 mmol/L l‐glutamine, 100 U/mL penicillin, 100 µg/mL streptomycin sulphate (Biofluids, Rockville, MD) and 20% foetal bovine serum (Equitech‐Bio, Kerrville, TX) at 37°C, under an atmosphere containing 5% CO_2_ and 1% O_2_. Early passages of MSC [1‐5] were used for this study. Cell viability was determined by trypan blue exclusion, and cell surface expressions of standard human MSC markers were previously reported.^21^

Approximately 2 hours after pFUS exposures, rats were given an IV injection of sodium nitroprusside (1 mg/kg; Roche) [4] followed immediately by 3 × 10^6^ human MSC in 300 μL of Hanks’ balanced saline solution (HBSS) containing 10 U/mL of sodium heparin.

### 2.5 Histological analyses

For histologic and immunohistochemical (IHC) evaluation, rats were perfused with 4% paraformaldehyde (PFA) in 1× phosphate‐buffered saline (PBS) following euthanasia and equilibrated in 30% sucrose following overnight fixation in 4% PFA. To identify the pFUS‐induced ischaemia, pimonidazole (60 mg/kg) (Hypoxyprobe, HPI Inc, MA) was intravenously infused 90 minutes before euthanasia. Frozen tissue was sectioned (10µm) and permeabilized with 0.1% Tween‐20 in 1X PBS (TPBS) followed by antigen retrieval using proteinase‐K solution (Life Technologies, Frederick, MD). Tissue sections were blocked with SuperBlock (Thermo Fisher Scientific, Waltham, MA) and were incubated with a primary antibody (10 µg/mL): anti‐rat albumin (Abcam), anti‐HIS48 (Bio‐Rad), CD68) (Abcam), anti‐pimonidazole (HPI Inc) or anti‐human mitochondria (Abcam) for 1 hour at room temperature. Slides were cover‐slipped using a mounting media containing DAPI and were scanned using a fluorescence slide scanner (Aperio FL, Leica Biosystem Inc, Buffalo Grove, IL). The fluorescent IHC (fIHC) was quantified by evaluating the pixel intensity in randomly selected multiple location within pFUS‐targeted region using ImageJ (National Institutes of Health) (5 sections/animal, n = 3). Haematoxylin and eosin (H&E) stain was also performed to evaluate macroscopic changes such as tissue abnormities and microhaemorrhage following sonication, and the tissue slides were scanned with a bright field scanner (Aperio SC2, Leica Biosystem Inc).

### 2.6 Statistical analyses

All data are presented as mean ± standard deviation, and data analyses were performed with Prism (version 7, GraphPad Software, Inc La Jolla, CA). One‐way analysis of variance (ANOVA) with Dunnett *post hoc* tests were used for multiple comparisons. Means were compared with two‐tailed unpaired *t* test. The *P*‐value less than 0.05 was considered as statistical significance.

## 3 RESULTS

### 3.1 MR‐guided pFUS

Rats were placed approximately 90 degree rotated to the left on the MR‐compatible pre‐clinical FUS system in order to pose the heart perpendicular to the ultrasonic transducer that could minimize the sonication to the lungs (Figure 1A and F). Under MR guidance, the left ventricle was sonicated with 3 MPa of PNP, which is approximately 1.6 MPa intramyocardially (equivalent to a MI = 1.7) as a result of attenuation at rat chest wall.^22^ Following pFUS treatment, no macroscopic and microscopic evidence of pulmonary damage was observed as has been previously reported at sonications at PNP = 6 MPa.^22^

### 3.2 Molecular responses following pFUS to the heart

**Figure 2:**
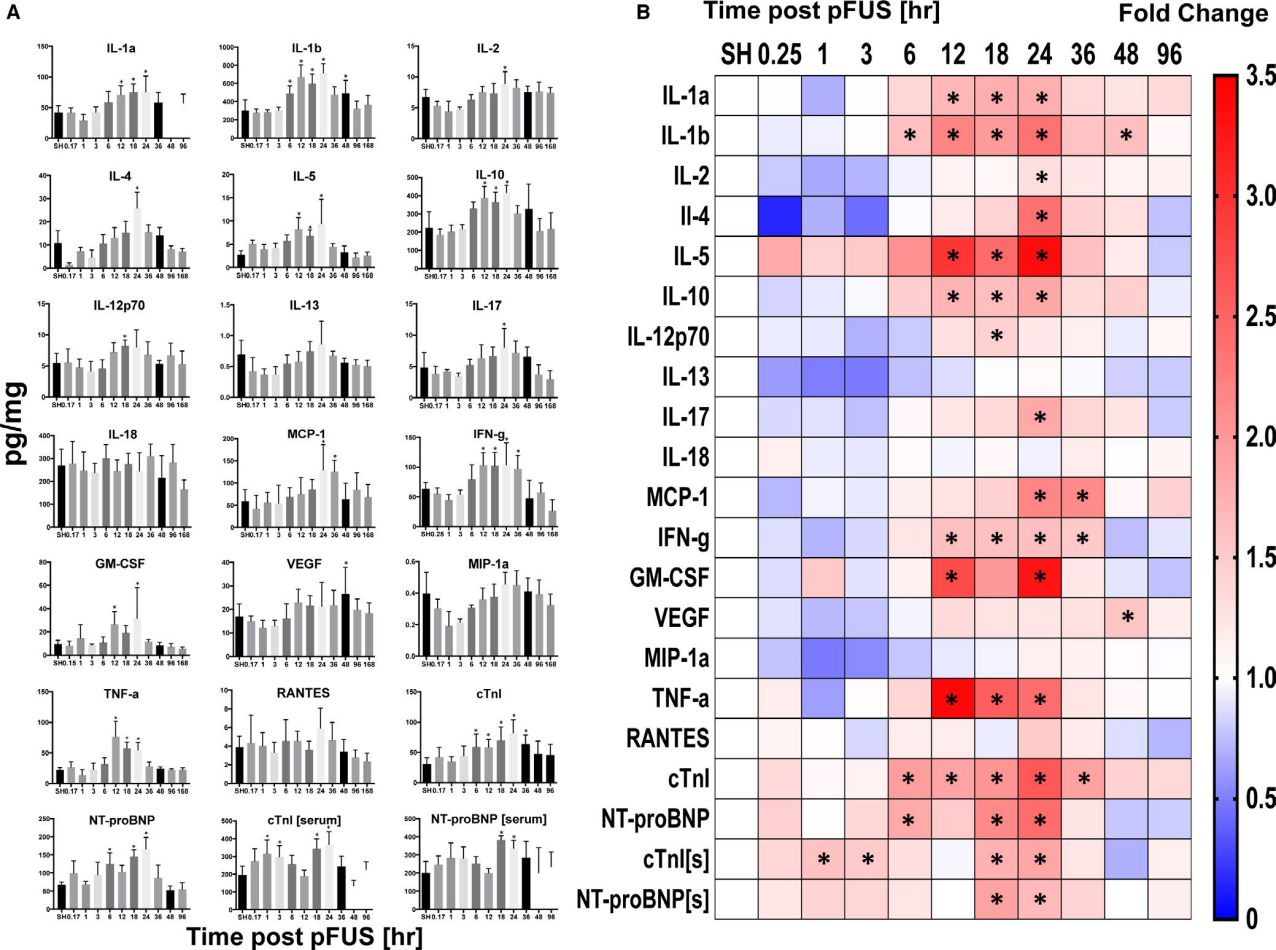
Molecular analysis of myocardial lysate and blood following pFUS to the heart (n = 4‐10/group). A, Time course activation of CCTFs and cardiac markers following pFUS treatment. The x axes represent the time [hour] post‐pFUS, and the y axes represent the concentration of CCTFs and cardiac markers in myocardium [pg/mg]. B, Heatmap (fold changes) of proteomic changes in pFUS‐treated myocardium over time. The levels of markers were quantified by ELISA and were normalized to SHAM control. Asterisks represent the statistical significance set at *P* < .05 based on ANOVA

Following pFUS at 3 MPa (n ≥ 5 rats per group), animals were euthanized at 0.25, 1, 3, 6, 12, 18, 24, 36, 48 and 96 hours. Sham‐sonicated (transducer power = 0 W) rats were euthanized and presented as the '0 hour' time point. Significant increases in CCTF were detectable starting at 6 hour post‐pFUS with increased expression of interleukin (IL)‐1β (*P* < .05, ANOVA) (Figure 2A and B). Between 12 and 24 hours post‐pFUS, significant (*P* < .05, ANOVA) increases in the expression of IL‐1α, IL‐2, IL‐5, IL‐12, IL‐17, monocyte chemoattractant protein‐1 (MCP‐1), interferon (IFN)‐γ, granulocyte macrophage colony‐stimulating factor (GM‐CSF) and TNF‐α were observed. During the time period, there was also significant increased expression of anti‐inflammatory cytokines such as IL‐4 and IL‐10 (Figure 2A and B). Vascular endothelial growth factor (VEGF) was significantly elevated (*P* < .05, ANOVA) at post‐48 hours of pFUS. All of sonication‐induced CCTF elevation returned to similar levels as sham‐treated controls by 48 hours.

The cardiac injury markers, cTnI and NT‐proBNP, were also evaluated in serum and tissue homogenates. In serum, significant (*P* < .05) increases in cTnI and NT‐proBNP were observed at 1 and 18 hours, respectively. Levels of both markers returned to the baseline levels by 36 hours post‐pFUS. In comparison, myocardial levels of both cTnI and NT‐proBNP were significantly increased (*P* < .05) between 6 and 36 hours following pFUS and returned to baseline levels by 48 hours (Figure 2A and B).

### 3.3 Histological analysis

**Figure 3:**
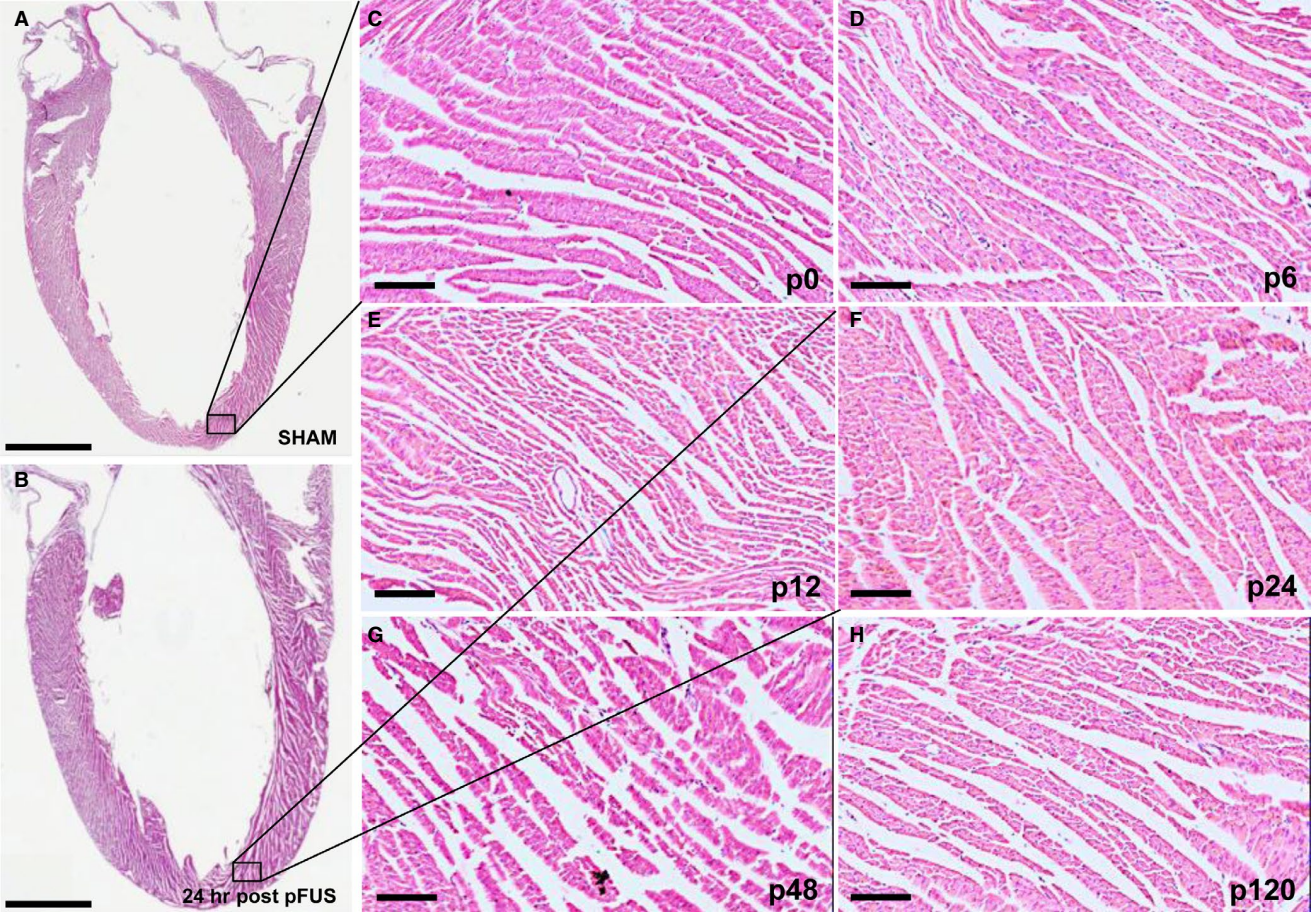
Time course of histologic evaluation of cardiac tissue following pFUS treatment (n = 3/group). A and B, Long axis LV sections of H&E staining in SHAM and 24 h post‐pFUS shows no detectable morphologic changes. Black boxes represent the regions of magnification. C‐H, Time course of morphologic analysis by H&E staining revealed that pFUS treatment did not result in macroscopic damages in apical myocardium. Scale bar = 2 mm (A‐B) and 100 µm (C‐H). C‐H represents Sham (p0), post‐6 (p6), post‐12 (p12), post‐24 (p24), post‐48 (p48) and post‐120 (p120) hrs at post‐pFUS, respectively

**Figure 4:**
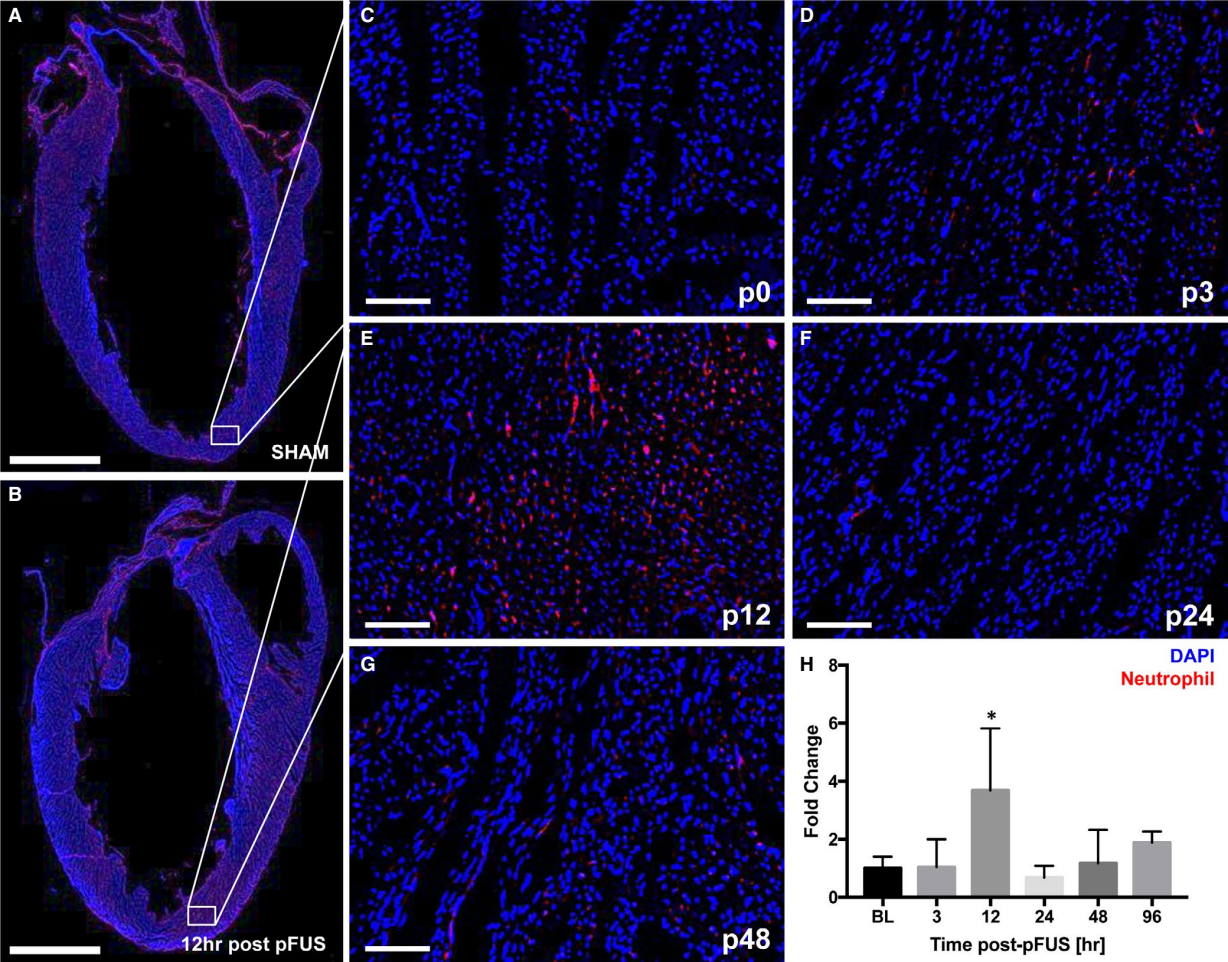
fIHCs of neutrophil infiltration in the pFUS‐targeted myocardium (n = 3/group). A and B, Long axis LV section of the fIHC in SHAM and post‐pFUS shows the significantly higher number of neutrophils (HIS48^+^ cells) was observed at post‐12 h compared with the SHAM. White box represents the region of interest in the heart. C‐G, Time course of neutrophil infiltration revealed that pFUS treatment resulted in significant number of neutrophils in pFUS‐targeted region at post‐12 h. H, Quantification of neutrophil positive staining in the ROI at difference time points. C‐G represents Sham (p0), post‐3 (p3), post‐12 (p12), post‐24 (p24) and post‐48 (p48) hours at post‐pFUS, respectively. Red colour represents the neutrophils (HIS48^+^ cells). Asterisks represent the statistical significance set at *P* < .05 based on ANOVA. Scale bar = 2 mm (A) and 100 µm (D‐M)

**Figure 5:**
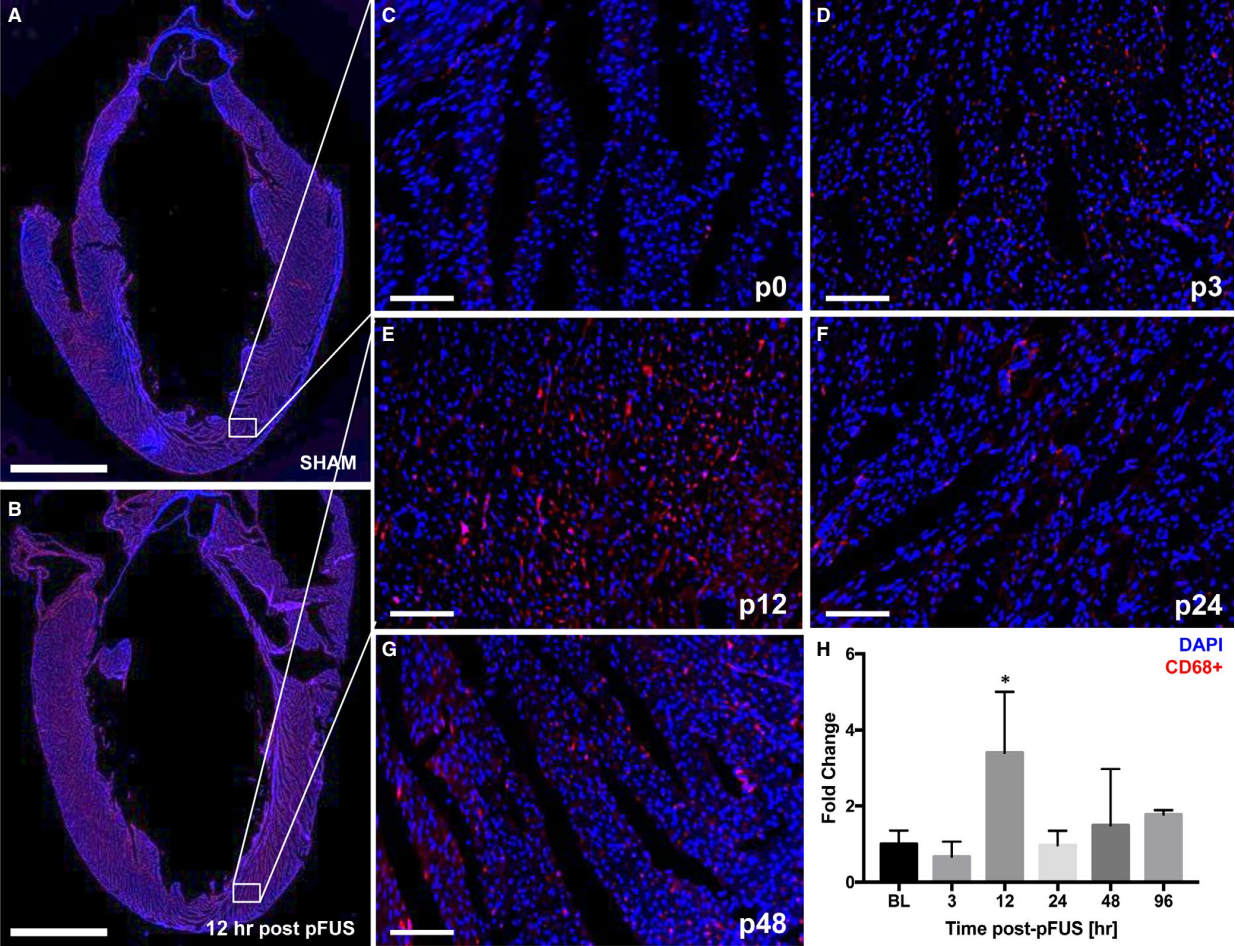
fIHC staining for CD68^+^ macrophages in the pFUS‐targeted myocardium (n = 3/group). A and B, Long axis LV section of fIHC in SHAM and post‐pFUS shows the significantly higher number of Macrophage (CD68^+^ cells) was observed at post‐12 h compared with the SHAM. White box represents the region of interest in the heart. C‐G, Time course of macrophage infiltration revealed that pFUS treatment resulted in significant number of macrophages in pFUS‐targeted region at post‐12 h. (H) There was a quantitative increase in fluorescence detected at 12 h post‐pFUS for macrophages. C‐G represents Sham (p0), post‐3 (p3), post‐12 (p12), post‐24 (p24) and post‐48 (p48) hours at post‐pFUS, respectively. Red colour represents the macrophage (CD68^+^ cells). Asterisks represent the statistical significance set at *P* < .05 based on ANOVA. Scale bar = 2 mm (A) and 100 µm (D‐M)

**Figure 6:**
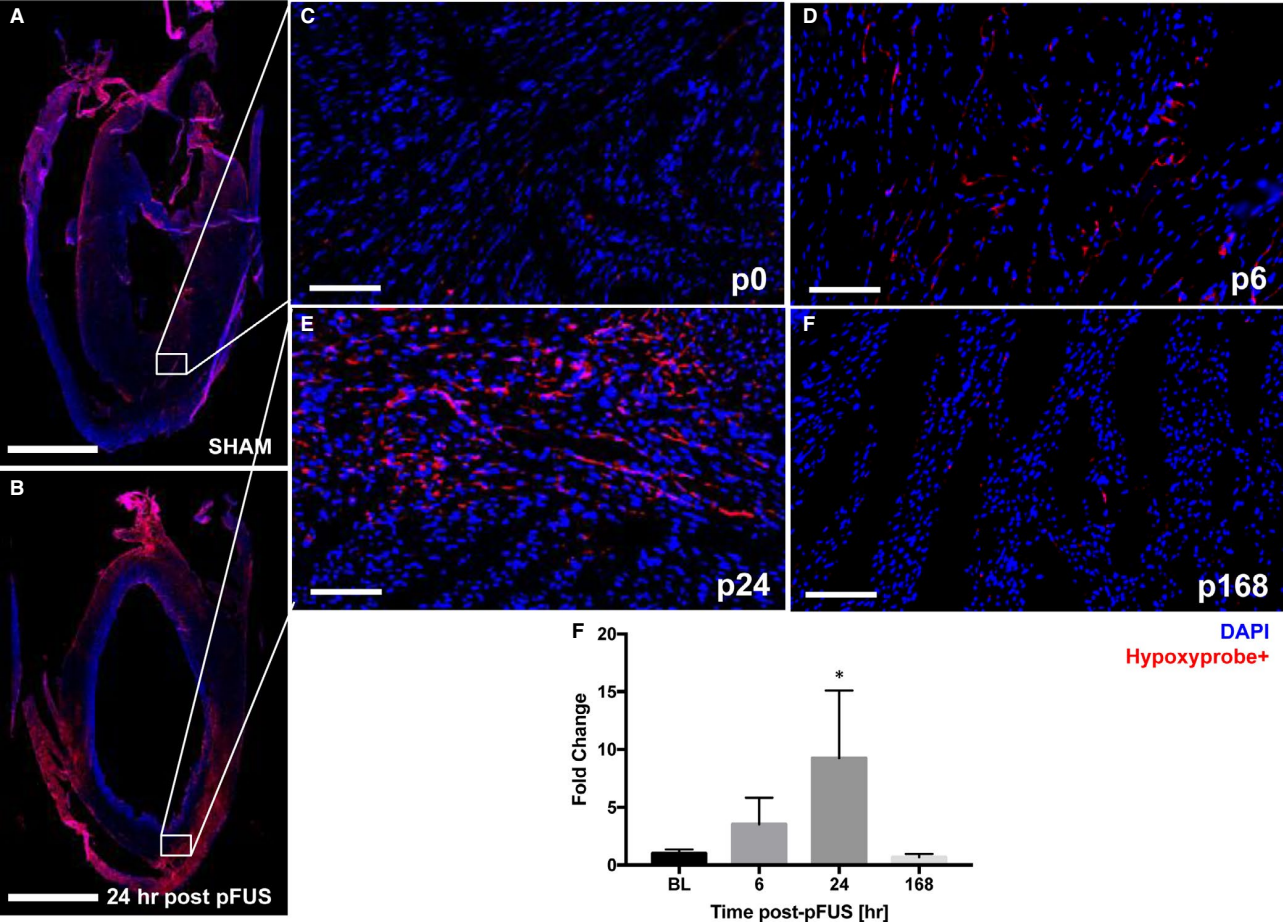
fIHCs of hypoxia staining (n = 3/group). A and B, Long axis LV section of fIHC staining. White boxes represent the ROI in SHAM (A) and post‐pFUS at 24 h (B). (C‐F) There was a heterogenous uptake of the hypoxia probe at 24 h post‐pFUS (E) compared with SHAM (C) and returned to baseline after 168 h (F). White box represents the region of interest in the heart. C‐F, Time course of hypoxia in the pFUS‐targeted myocardium. G, Quantification of areas of hypoxia staining in the multiple pFUS‐targeted region. C‐F represents Sham (p0), post‐6 (p6), post‐24 (p24) and post‐168 (p168) hours at post‐pFUS, respectively. Red colour represents the hypoxia (Hypoxyprobe^+^ cells). Asterisks represent the statistical significance set at *P* < .05 based on ANOVA. Scale bar = 2 mm (A and B) and 100 µm (C‐H)

**Figure 7:**
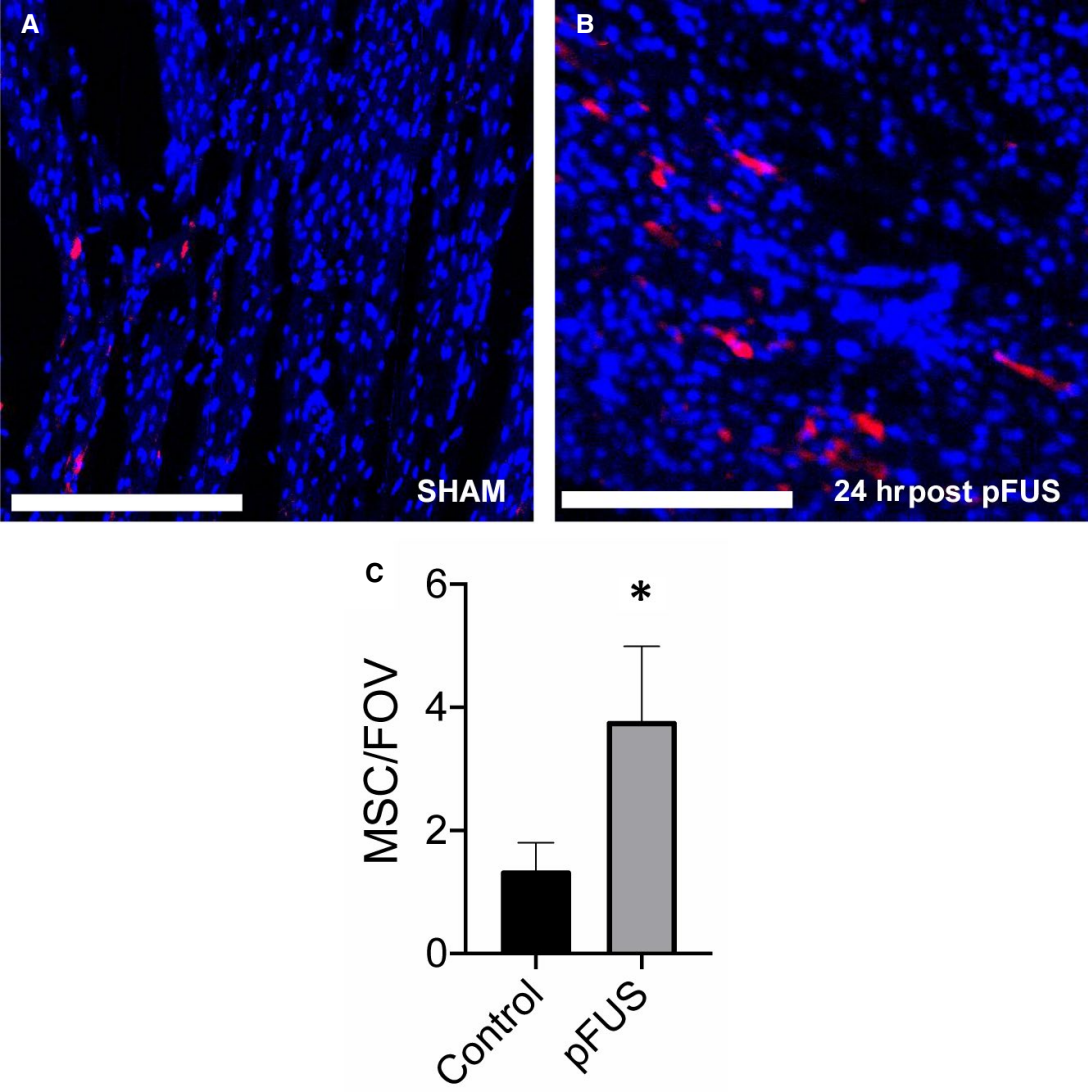
fIHC of anti‐human mitochondrial staining of pFUS targeted and controls rats (n = 3/group) following 24 h after infusion of human MSC. A and B, the ROI of the SHAM and pFUS‐targeted myocardium, respectively. C, Quantitative analysis of numbers of human MSC (red) in rat myocardium compared with controls. Asterisks represent the statistical significance set at *P* < .05 based on ANOVA. Scale bar = 300 µm

To evaluate the morphologic changes in pFUS‐targeted myocardium, H&E staining was performed on hearts harvested at various time points of post‐pFUS. There were no discernable differences between sham‐treated controls and pFUS‐treated hearts on H&E sections over 120 hours post‐sonication. There was no evidence of oedema or microhaemorrhage in the myocardium at any time point (Figure 3). IHC analyses revealed significant infiltration (*P* < .05) of neutrophils (HIS48^+^ cells) and macrophages (CD68^+^ cells) in treated myocardium compared with sham‐treated hearts (Figure 4). Increases in both neutrophils (Figure 4) and macrophages (Figure 5) were detected within the first 12 hours following sonication and cleared by 24 hours. IHC staining for pimonidazole, which was infused prior to euthanasia, revealed increased areas of hypoxic myocardium in the sonicated hearts at 24 hours (Figure 6) post‐sonication compared with sham‐treated control animals. Another cohort of rats (n = 3 per group) was treated with pFUS followed by IV infusions of human MSC 2 hours post‐pFUS (Figure 7). At 24 hours post‐pFUS, animals were euthanized and IHC for MSCs (human mitochondria^+^ cells) revealed a 2.5‐fold increase in MSC residing in sonicated myocardium compared with sham‐treated rats.

## 4 DISCUSSION

MR‐guided pFUS is a non‐invasive tool for modulating tissue microenvironments and has potential clinical translational in the treatment of cardiac diseases. In this study, pFUS mechanotransductive effects were evaluated based on the molecular changes in the myocardium and the ability to enhance homing permeability and retention (EHPR) of infused MSC to targeted regions.^5^, ^6^, ^7^, ^8^, ^17^, ^18^, ^19^, ^23^ MRI guidance for pFUS provided the ability for accurate targeting to the volumes of interest (VOI) in the myocardium that ultimately resulted in the induced molecular responses consistent with increased expression of pro‐ and anti‐inflammatory CCTF between 6 and 36 hours.^4^, ^5^, ^6^, ^7^, ^8^, ^22^, ^24^ In comparison with the current study, we previously reported that targeted pFUS to rat myocardial septum with higher PNP of 6 MPa induced immediate (within 0.17 hour) increases in pro‐inflammatory CCTF that were not associated with changes in cardiac biomarkers or evidence of myocardial damage. However, pFUS at 6 MPa was associated with substantial pulmonary haemorrhage.^22^ As a result of this observation, we decreased pFUS PNP of 3 MPa in an effort to reduce lung damage. pFUS targeted to the myocardium at lower intensities resulted in a delayed proteomic response in increased expression of CCTF starting at ~6 hours without evidence of pulmonary haemorrhage. The delayed molecular responses in the sonicated heart was associated with increased IL‐1β and peaked in the number of significant CCTF occurring at 24 hours. The increase in IL‐1β at 6 hours could suggest transient cardio‐myofibril damage associated with sterile inflammation through an NF‐κB pathway.^25^ Moreover, significant increases in pro‐inflammatory factors such as TNF‐α, IFN‐γ, IL‐1α, IL‐1β, IL‐2 and MCP‐1 occurred within 24 hours. The activation of TNF‐α, IL‐1β and other chemotactic factors (GM‐CSF and MCP‐1) could also be associated with intrinsic self‐remodelling of myocardium responding to mechanical stretch induced by pFUS.^26^, ^27^, ^28^ Significant increases in IL‐4 and IL‐10 were also detected and could have been responsible for the lack of inflammatory damage in the myocardium. The increase in VEGF at 48 hours could also contribute the repair to vascular disruptions and be driven by the transient ischaemic response following pFUS. Interestingly, the CCTF responses in myocardium were similar to those previously observed in kidney and skeletal muscle.^4^, ^5^, ^6^, ^7^, ^8^, ^10^, ^29^

There are several reports evaluating the expression of cardiac biomarkers, such as creatine kinase MB, heart‐type fatty acid‐binding protein (H‐FABP), TNF‐a, cTnI and NT‐proBNP from the blood following cardiac contusion.^30^, ^31^, ^32^, ^33^ However, there are few reports examining the cardiac biomarker expression directly in the myocardium. We performed a serial time course to evaluate the cardiac biomarkers, cTnI and NT‐proBNP, in both pFUS‐targeted myocardium and serum. The findings of increased cTnI and NT‐proBNP suggest pFUS in this study probably resulted in mild mechanical stress to the LV as it has been associated with regular exercise, which has been shown to elevate cardiac injury markers.^34^

Histological examination of the pFUS‐targeted myocardium revealed no macroscopic or microscopic changes on H&E staining over 120 hours. Consistent with the observed molecular changes in the myocardial microenvironment, neutrophil and macrophage infiltration into the extracellular spaces at 12 hours post‐pFUS would further support the presence of an inflammatory response within the myocardium. The transient increase presences of inflammatory cells in the sonicated myocardium coincided with significant increases in pro‐inflammatory CCTF (eg sTNFα, IL1α, IL1β, IL5, INFγ) and the presence of cTnI.

Pimonidazole staining to detect hypoxia revealed only in the targeted regions at 24 hours post‐pFUS and returned to baseline levels by 168 hours. These results suggest that pFUS induced transient areas of hypoxia that coincided with the peak increased expression of pro‐inflammatory CCTF in the myocardium. Tissue hypoxia was not investigated in previous reports. However, even mild and transient hypoxia drives numerous molecular signalling pathways in tissues that could contribute to the EHPR effect of pFUS on MSC tropism to targeted tissues. Further investigation into oxygen status of tissues following pFUS should be undertaken whether it influenced the increased MSC tropism to the myocardium as a result of hypoxia and molecular signalling cascades.^35^, ^36^, ^37^, ^38^ Moreover, the effects of pFUS to induce a sterile inflammatory response in the targeted myocardium does not lead to persistent tissue damage require further examination.^39^, ^40^

There are several studies demonstrating the potential use of ultrasound (both focused and unfocused) to the heart coupled with MB infusion that generated molecular changes in the myocardium increased tropism of systemically infused stem cells.^16^, ^17^, ^18^ It has been reported that pFUS + MB followed by infusion of endothelial progenitor cells in a model of chronic cardiomyopathy in the guinea pig resulted in improvement in LVEF and myocardial perfusion at 20 weeks post‐sonication.^19^ In acute myocardial infarction rat model, the combination of pFUS + MB results in increased expression of IL‐1β, 4, 6, MCP‐1 and TNF‐α within 15 minutes post‐sonication.^17^ In that study, intracoronary artery infusion of MSC resulted in increased homing of cells to infarcted myocardium. These studies reported between 2 and 5 times more stem cells in the sonicated myocardium at euthanasia. However, these studies intentionally employ UTMD to mediate bioeffects. MB destruction is well known to cause profound tissue damage and may be unsuitable for regenerative medicine strategies. Although the two different sources of mechanical forces, either from UTMD or direct pFUS, would not be directly compared, we believe that the underlying molecular mechanism that attracts the MSC homing would be similar. In the current study, pFUS induced an EHPR effect on IV‐infused human MSC that led the significantly increased numbers of human cells homing to the targeted regions by 24 hours. These results are similar to our previous findings^17^, ^18^ in which the increased tropism to murine skeletal muscle and kidney in response to the release of chemoattractants (ie MCP1, GM‐CSF) following pFUS at 4 MPa.^4^, ^5^, ^6^, ^7^, ^8^, ^22^, ^24^, ^25^, ^26^, ^27^, ^28^, ^30^, ^31^, ^32^, ^33^, ^39^, ^40^ These results are consistent with previous observation, which demonstrated the ability of pFUS to induce changes in the tissue microenvironment that would EHPR of infused stem cells into targeted regions of hypoxia.^13^ We speculate that MSC homing plays a major role in paracrine signalling rather than in differentiation to myocardium muscle. Future investigations would be necessary to understand whether the induced MSC homing and hypoxia in the pFUS‐targeted region modulate the inflammatory responses through the paracrine signalling in areas of myocardial infarction that potentially can result in improved clinical outcomes.

There are several limitations of this study that need to be addressed. MR‐guided pFUS was performed without cardiac or respiratory gating, as a result of the technical limitations interfacing the MRI and focused ultrasound instrumentation. As a result, the pFUS‐targeted area in the myocardium may have been larger than intended and we cannot account for off target effects or placing the US beam within the ventricular cavity during parts of the cardiac cycle. Moreover, the MSC that were used in this study were expanded from volunteers who had bone marrow biopsies. However, it is possible that more efficient homing and survival capacity may be achieved by using stem cells derived from rats that would obfuscate an immune response and result in prolonged in vivo lifespan of MSC.^41^, ^42^ Moreover, we did not examine the possible changes in cellular metabolism such as functional changes by mitochondria induced by pFUS.^18^ Future studies will assess cardiac functional changes following pFUS including optimization of sonication parameters to induce necessary MSC tropism without elevating injury biomarkers. The results of this study provide a basis for additional investigation of treatment parameters that could be translatable as part of a regenerative medicine strategy in the treatment of cardiac injury or disease.

## CONFLICT OF INTEREST

The authors declare no conflict of interests.

## AUTHOR CONTRIBUTIONS

KWJ, SRB and JAF: Study design; KWJ, TT, BKL, RBR and SRB: Experiments; KWJ, TT, RR, SRB and JAF: Data analysis and interpretation; KWJ, SRB and JAF: Manuscript drafting. All authors: Manuscript reviewing.

## Data Availability

The data that support the findings of this study are available on request from the corresponding author, KWJ The data are not publicly available.
